# Brain rhythmic abnormalities in convalescent patients with anti-NMDA receptor encephalitis: a resting-state EEG study

**DOI:** 10.3389/fneur.2023.1163772

**Published:** 2023-07-20

**Authors:** Dengchang Wu, Lin Jiang, Runyang He, Baodan Chen, Dezhong Yao, Kang Wang, Peng Xu, Fali Li

**Affiliations:** ^1^Department of Neurology, The First Affiliated Hospital, School of Medicine, Zhejiang University, Hangzhou, China; ^2^The Clinical Hospital of Chengdu Brain Science Institute, MOE Key Lab for Neuroinformation, University of Electronic Science and Technology of China, Chengdu, China; ^3^School of Life Science and Technology, Center for Information in BioMedicine, University of Electronic Science and Technology of China, Chengdu, China; ^4^Research Unit of NeuroInformation, Chinese Academy of Medical Sciences, Chengdu, China; ^5^School of Electrical Engineering, Zhengzhou University, Zhengzhou, China; ^6^Radiation Oncology Key Laboratory of Sichuan Province, Chengdu, China

**Keywords:** anti-NMDAR encephalitis, EEG, power spectral density, brain network, clinical assessment

## Abstract

**Objective:**

Anti-N-methyl-D-aspartate receptor encephalitis (anti-NMDARE) is autoimmune encephalitis with a characteristic neuropsychiatric syndrome and persistent cognition deficits even after clinical remission. The objective of this study was to uncover the potential noninvasive and quantified biomarkers related to residual brain distortions in convalescent anti-NMDARE patients.

**Methods:**

Based on resting-state electroencephalograms (EEG), both power spectral density (PSD) and brain network analysis were performed to disclose the persistent distortions of brain rhythms in these patients. Potential biomarkers were then established to distinguish convalescent patients from healthy controls.

**Results:**

Oppositely configured spatial patterns in PSD and network architecture within specific rhythms were identified, as the hyperactivated PSD spanning the middle and posterior regions obstructs the inter-regional information interactions in patients and thereby leads to attenuated frontoparietal and frontotemporal connectivity. Additionally, the EEG indexes within delta and theta rhythms were further clarified to be objective biomarkers that facilitated the noninvasive recognition of convalescent anti-NMDARE patients from healthy populations.

**Conclusion:**

Current findings contributed to understanding the persistent and residual pathological states in convalescent anti-NMDARE patients, as well as informing clinical decisions of prognosis evaluation.

## 1. Introduction

As the most frequent human autoimmune encephalitis, anti-N-methyl-D-aspartate receptor encephalitis (anti-NMDARE) is characterized by diverse psychiatric and neurological features, such as memory impairment and psychosis (1, 2). In clinical practice, in addition to the evaluation from experienced physicians, the detection of IgG antibodies against the GluN1 subunit of the receptor in the serum and cerebrospinal fluid (CSF) has long been the golden criterion for anti-NMDARE diagnosis (3, 4). Whereas the follow-up of antibody titers was not perfectly correlated with the disease course (5), antibodies may still be recognized in the CSF and serum of patients even after clinical recovery (6). Although 75% of the patients have a favorable outcome with substantial recovery (7), persistent deficits in memory, attention, and executive functioning (8–12) still occur.

The diagnosis based on magnetic resonance imaging can be normal or nonspecific (13), providing limited diagnostic and prognostic value. Given a quantitative and specific method is lacking to guide noninvasive and precise evaluation for this disease, other objective tests seem to be imperative to track therapy with a specific biomarker of brain activity, as well as to potentially monitor the treatment course. In this regard, the registration of spontaneous activity by electroencephalogram (EEG) seems to be a practical way, which has been extensively incorporated into clinical evaluations for its inexpensive, non-invasive, and widely available nature. EEG is abnormal in almost anti-NMDARE patients during the acute phase, showing disorganized background activity (1, 14), which seems to be useful in the clinical diagnosis of anti-NMDARE (15). Whereas the majority of previous EEG research concentrates on the raw signal manifestations during the acute stage (16, 17), further exploring the EEG biomarkers may have the potential for rapid diagnosis and prognostic evaluation of anti-NMDARE within varying courses. More importantly, scalp EEG provides a noninvasive and objective tool for studying the brain (dys)function (18, 19); thus, EEG is believed to be capable to assess the brain deficits of anti-NMDARE patients even during convalescence, as well as to differentiate convalescent patients from healthy controls (HCs).

Hence, based on our previous studies which verified the negative effect of anti-NMDARE on the patient’s brain at different therapeutic stages, such as hyperactivated regional activity in hippocampus/parahippocampus and worse memory retrieval performance, etc. at the post-acute stage (20, 21), in the current study, quantitative EEG analysis was carried out to probe the role of the brain rhythmic alteration in the course of the anti-NMDARE. Therein, we expected that the distinguished resting-state EEG metrics could be regarded as the biomarkers to quantify the therapy of anti-NMDARE and provide potential clinical guidance for the future follow-up of these patients. To achieve this, resting-state EEG datasets of anti-NMDARE patients during the convalescence and gender- and age-matched HCs were collected. Theoretically, cortical oscillations play essential roles in a wide range of neural activities, especially since changes in spectral power by neurocognitive disease within specific rhythms have been wildly reported (22, 23). In addition, other than isolated brain regions, the network that helps reveal information propagation among spatially distributed regions (24) was also considered. As such, power spectral density (*PSD*) which provides valuable information on regional power characteristics of spontaneous activities was first investigated, and brain network analysis was performed to explore the intrinsic rhythmic difference between anti-NMDARE patients and HCs, which is expected to provide potential biomarkers for distinguishing convalescent anti-NMDARE patients from HCs, as well as tracking the therapeutic effect in future studies.

## 2. Materials and methods

### 2.1. Patients

Under the approval of the Ethics Committee of the First Affiliated Hospital, Zhejiang University School of Medicine, 36 right-handed participants, including 18 convalescent anti-NMDARE patients (15 females, age 27.22 ± 12.28, Table 1) and 18 gender- and age-matched HCs (15 females, age 27.56 ± 11.51), were enrolled. Herein, all patients were diagnosed according to the detection of IgG antibodies for NMDA receptors and other typical clinical symptoms (1, 4). And in addition to the history of neurologic or psychiatric disorders, we also excluded the patient with abnormal lesions in magnetic resonance imaging (MRI) scans to exclude the possible coexisting conditions. Concerning the HC, these gender- and age-matched participants had been also confirmed to have no history of psychological or neurological disorders. Before resting-state EEG monitoring, all participants had fully understood the experimental protocol and signed the written informed consent.

**Table 1:** Clinical and demographic information of the patients.

Participant	Sex	Age	NMDAR-IgG onset	NMDAR-IgG recovery phase	mRS onset	mRS recovery
Patient 1	F	50	1:32	1:1	5	1
Patient 2	F	32	1:32	1:1	5	0
Patient 3	F	15	1:32	1:1	5	0
Patient 4	F	22	1:32	1:10	5	0
Patient 5	F	25	1:32	1:1	2	0
Patient 6	F	20	1:32	1:10	3	0
Patient 7	F	24	1:32	1:1	5	0
Patient 8	M	46	1:3.2	0	5	1
Patient 9	M	28	1:32	1:10	5	0
Patient 10	M	58	1:32	1:1	5	0
Patient 11	F	22	1:32	1:10	5	0
Patient 12	F	15	1:10	1:1	5	0
Patient 13	F	17	1:10	1:1	5	0
Patient 14	F	29	1:32	1:1	5	0
Patient 15	F	19	1:32	1:10	5	0
Patient 16	F	21	1:10	1:1	5	0
Patient 17	F	29	1:32	1:10	5	0
Patient 18	F	18	1:32	1:1	5	0

### 2.2. Clinical status

Patients can be diagnosed as stronger positive, positive, weak positive, and negative based on the antibody titers. And the modified Rankin Scale (mRS) was also measured to evaluate the neurological outcome per patient. In essence, patients are considered fully recovered (i.e., mRS = 0) if they can return to work; patients are considered mildly deficient (i.e., mRS = 1 or 2) if they can return to most daily activities and keep stable for more than 2 months; in other cases, patients were considered seriously deficient. Herein, all patients enrolled were considered in convalescence, as the antibody titer level was negative and the maximal mRS score was 1 according to the medical records (Table 1). In the meantime, we tested 8–18 antibodies of autoimmune encephalitis in the CSF and serum for all patients, and all of them showed only NMDA-IgG positive.

### 2.3. EEG data recording

The resting-state EEG was collected by 32-channel digital video-EEG systems (Nicolet v32, Natus Neurology Incorporated, Middleton, WI, USA). All electrodes were positioned in compliance with the international 10/20 system. The impedance of each electrode was kept below 5 kΩ, and electrode AFz was set as the reference. EEG was band-pass filtered at 0.01–100 Hz and sampled at 500 Hz.

### 2.4. EEG analysis

**Figure 1:**
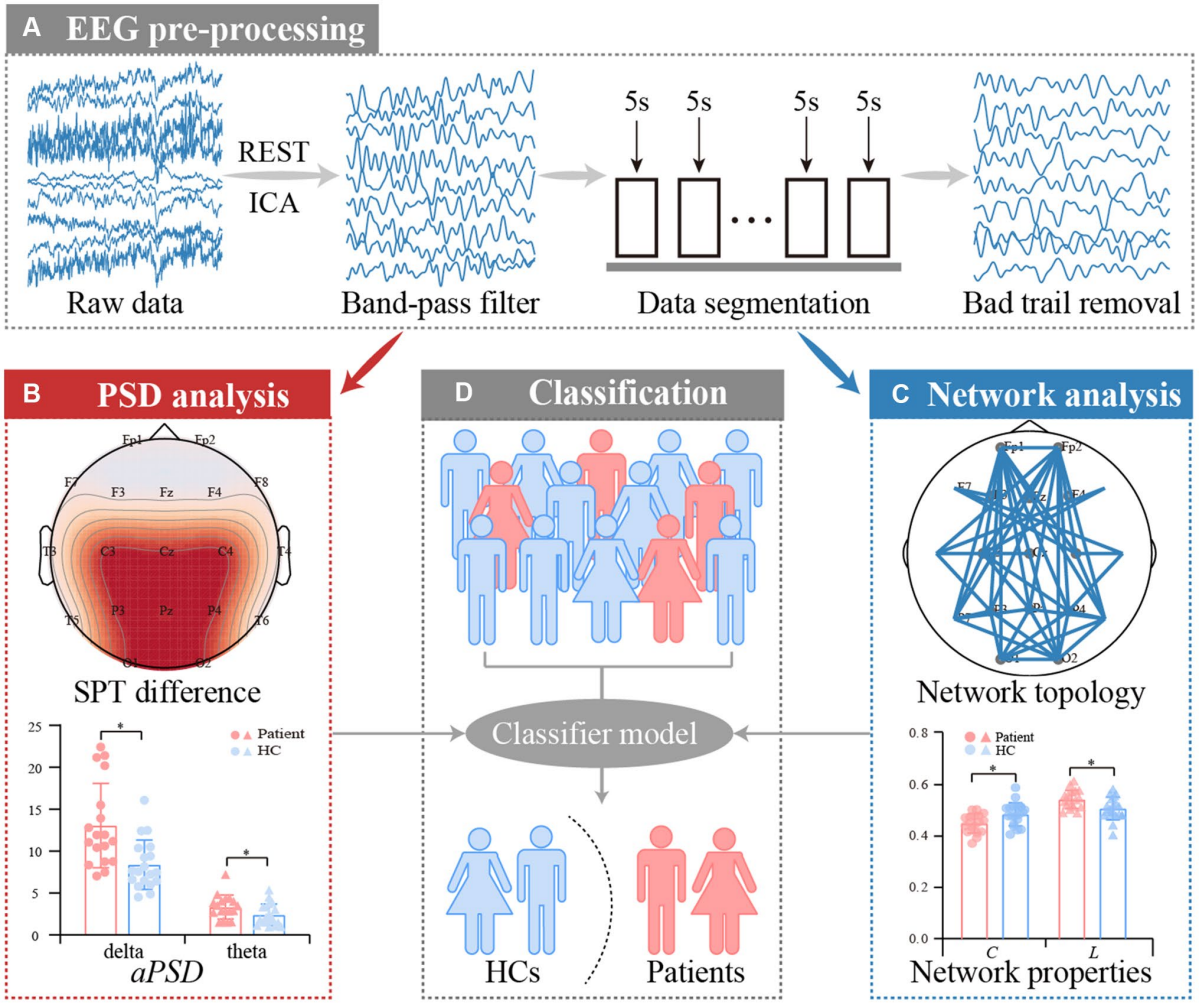
Analysis procedures for resting-state EEG datasets of anti-NMDARE patients. **(A)** EEG pre-processing, **(B)** *PSD* analysis, **(C)** PLV network analysis, and **(D)** Classification.

The analytical procedures are depicted in Figure 1 and consist of EEG pre-processing, *PSD* analysis, network analysis, and classification. The pre-processing aims to acquire artifact-free segments, and the following analyses will further explore the disease-induced aberrant patterns of resting-state rhythmic activity in convalescent patients. The details are described below.

#### 2.4.1. EEG pre-processing

The EEG datasets were first re-referenced to a neutral reference by the Reference Electrode Standardization Technique (REST) (25, 26). Thereafter, multiple standard procedures including the independent component analysis (27), 0.5–45 Hz bandpass filtering, 5 s data segmenting, and ±90 μV artifacts removal were carried out accordingly.

#### 2.4.2. Power spectral density

For each participant, the remaining segments after preprocessing were trial-averaged, and the corresponding *PSD* was then calculated by Welch’s method within a frequency range of interest *f*, i.e., delta (0.5–4 Hz), theta (4–8 Hz), alpha (8–13 Hz), beta (13–30 Hz), and gamma (30–45 Hz). Subsequently, the scalp power topographies (*SPT*s) per band were plotted, and the absolute *PSD* (*aPSD*) was also evaluated. Specifically, based on the *PSD* of each frequency bin *f*, the *aPSD* value was defined and calculated as,


(1) $$aPSD_{\xi }\left(\sigma \right)=\sum_{f=f_{1}}^{f_{2}}PSD\left(f\right),if\{\begin{array}{c} \sigma ==delta\\ \sigma ==\text{theta}\\ \sigma ==alpha\\ \sigma ==beta\\ \sigma ==gamma \end{array},then\{\begin{array}{c} \left[f_{1},f_{2}\right]=\left[0.5,\;4\right]\\ \left[f_{1},f_{2}\right]=\left[4,\;8\right]\\ \left[f_{1},f_{2}\right]=\left[8,\;13\right]\\ \left[f_{1},f_{2}\right]=\left[13,\;30\right]\\ \left[f_{1},f_{2}\right]=\left[30,\;45\right] \end{array}$$

where *ξ* denotes the types of anti-NMDARE patients or HCs.

#### 2.4.3. PLV network

Based on the preprocessed EEG segments, the phase-locking value (PLV) was then applied to construct related functional brain networks (28). First, to assess the instantaneous phases, *φ_u_*(*t*) and *φ_v_*(*t*), of signals *u*(*t*) and *v*(*t*), the Hilbert transform (*HT*) was applied to establish the analytical signal *H*(*t*),


(2) $$\begin{array}{l} H_{u}=u\left(t\right)+iHT_{u}\left(t\right)\\ H_{v}=v\left(t\right)+iHT_{v}\left(t\right) \end{array}$$

where *HT_u_*(*t*) and *HT_v_*(*t*) indicate the *HT* of the two signals and can be formulated as,


(3) $$\begin{array}{l} HT_{u}=\frac{1}{\pi }P.V.\int_{-\infty }^{\infty }\frac{u\left(t^{\prime }\right)}{t-t^{\prime }}dt^{\prime }\\ HT_{v}=\frac{1}{\pi }P.V.\int_{-\infty }^{\infty }\frac{v\left(t^{\prime }\right)}{t-t^{\prime }}dt^{\prime } \end{array}$$

where the *P.V.* is the Cauchy principal value. Consequently, the phases of the analytical signals, *φ_u_*(*t*) and *φ_v_*(*t*), are defined as,


(4) $$\begin{array}{l} \varphi_{u}=\arctan\frac{HT_{u}\left(t\right)}{u\left(t\right)}\\ \varphi_{v}=\arctan\frac{HT_{v}\left(t\right)}{v\left(t\right)} \end{array}$$

Then, the PLV can be calculated as,


(5) $$w^{PLV}=\left|\frac{1}{N}\sum_{j=0}^{N-1}e^{i\left(\varphi_{u}\left(j\Delta t\right)-\varphi_{v}\left(j\Delta t\right)\right)}\right|$$

where *N* and *t* indicate the sample number and the time point, respectively. *Δt* denotes the sampling period, *j* is the *j-*th sample point, and *w^PLV^* represents the connection weight.

For each interested band, the weighted adjacency matrixes were formed to index the information exchange among all electrodes, and then matrices of all segments were averaged to obtain the final matrix for each participant.

#### 2.4.4. Network properties

The characteristic path length (*L*) and clustering coefficient (*C*) were two essential network indicators reflecting the global and local information processing ability of brain networks, respectively, which can be computed by the brain connectivity toolbox (BCT, http://www.nitrc.org/projects/bct/) (29) as,


(6) $$C=\frac{1}{M}\sum_{i\in \theta }\frac{\sum_{j,l\in \theta }{\left(w_{ij}w_{il}w_{jl}\right)}^{1/3}}{\sum_{j\in \theta }w_{ij}\left(\sum_{j\in \theta }w_{ij}-1\right)}$$

(7) $$L=\frac{1}{M}\sum_{i\in \theta }L_{i}=\frac{1}{N}\sum_{i\in \theta }\frac{\sum_{j\in \theta ,j\neq i}d_{ij}}{N-1}$$

where *w_ij_* and *d_ij_* represent the connection strength and the shortest weighted path length between node *i* and *j*, respectively. *θ* and *M* indicate the set of all nodes and the node number of the network, respectively.

#### 2.4.5. Statistical analysis

Within each interested band, the independent sample *t*-test was statistically performed to quantify potential relationships, i.e., the differences in *SPT*, *aPSD*, network topologies, and properties, between the convalescent patients and HCs. Then, a false discovery rate (FDR) was performed to correct the *p*-values, in which the corrected significant *p*-values were verified to have at least *p* < 0.01.

#### 2.4.6. Classification between the anti-NMDARE patients and HCs

Considering that *PSD* and functional networks reflect different aspects of spontaneous brain operation, both might provide complementary and comprehensive information for discrimination between anti-NMDARE patients and HCs. In this work, in addition to *aPSD* and network properties, the discriminative spatial pattern of the network (SPN) was also calculated (30). Hence, using the SPN features, *aPSD*, and network properties, we further classified the convalescent patients from HCs by the support vector machine (SVM) with leave-one-out cross-validation (LOOCV) strategy (31). Thereafter, to evaluate the classification performance, the accuracy, specificity, and sensitivity were calculated as,


(8) $$\text{Accuracy}=\frac{n_{PAs}+n_{HCs}}{N_{PAs}+N_{HCs}}\times 100\%$$

(9) $$\text{Sensitivity}=\frac{n_{PAs}}{N_{PAs}}\times 100\%$$

(10) $$\text{Specificity}=\frac{n_{HCs}}{N_{HCs}}\times 100\%$$

where *n_PAs_* and *n_HCs_* represent the correct number of anti-NMDARE patients and HCs, respectively. *N_PAs_* and *N_HCs_* represent the total number of anti-NMDARE patients and HCs, respectively.

## 3. Results

### 3.1. Differential scalp power topographies

**Figure 2:**
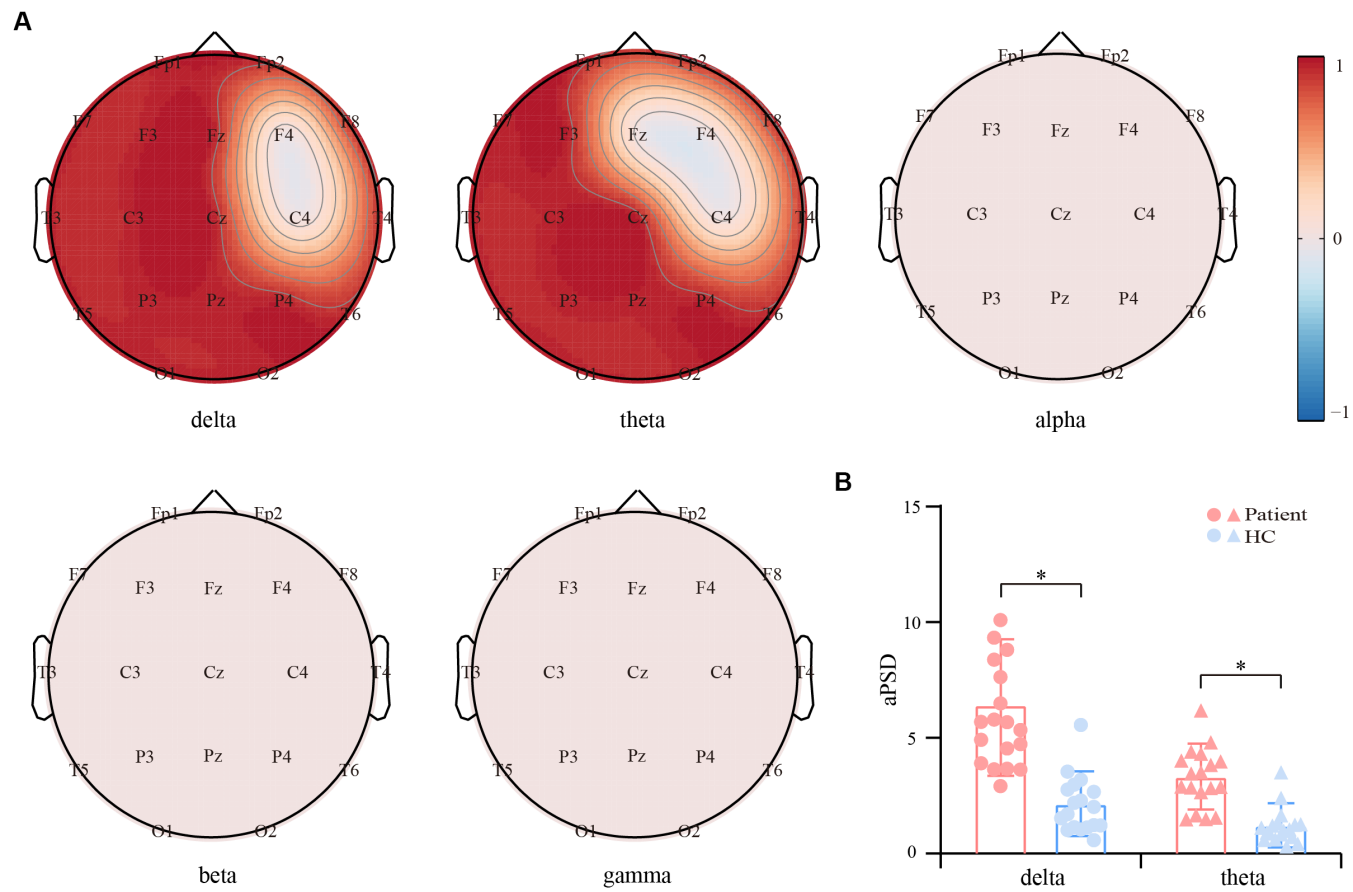
Differential resting-state power spectrum density (*PSD*) between the convalescent patients and HCs. **(A)** Scalp topographies of *PSD* differences within the five bands. Red and blue regions represent the enhanced and suppressed EEG power of the patients compared to that of the HCs (*p* < 0.01, FDR corrected), respectively. **(B)** Differential *aPSD* within the delta and theta bands. The blue and red bars indicate the HCs and patients, respectively. The symbol * represents *p* < 0.01.

Figure 2A exhibits the *SPT* patterns between convalescent patients and HCs in the five bands. Generally, in contrast to HCs, the EEG power of the convalescent patients experienced a significant enhancement within delta and theta bands (*p* < 0.01, FDR corrected), which were distributed in extensive frontal, parietal, and occipital regions. However, no differences were captured for the other three bands. Given the prominent variation of delta and theta *SPT*, the grand-averaged *aPSD* across all electrodes was calculated and statistically compared between both groups. As shown in Figure 2B, within both rhythms, the convalescent patients exhibit significantly increased *aPSD*, when compared to that of the HCs (*p* < 0.01).

### 3.2. Differential functional brain networks

**Figure 3:**
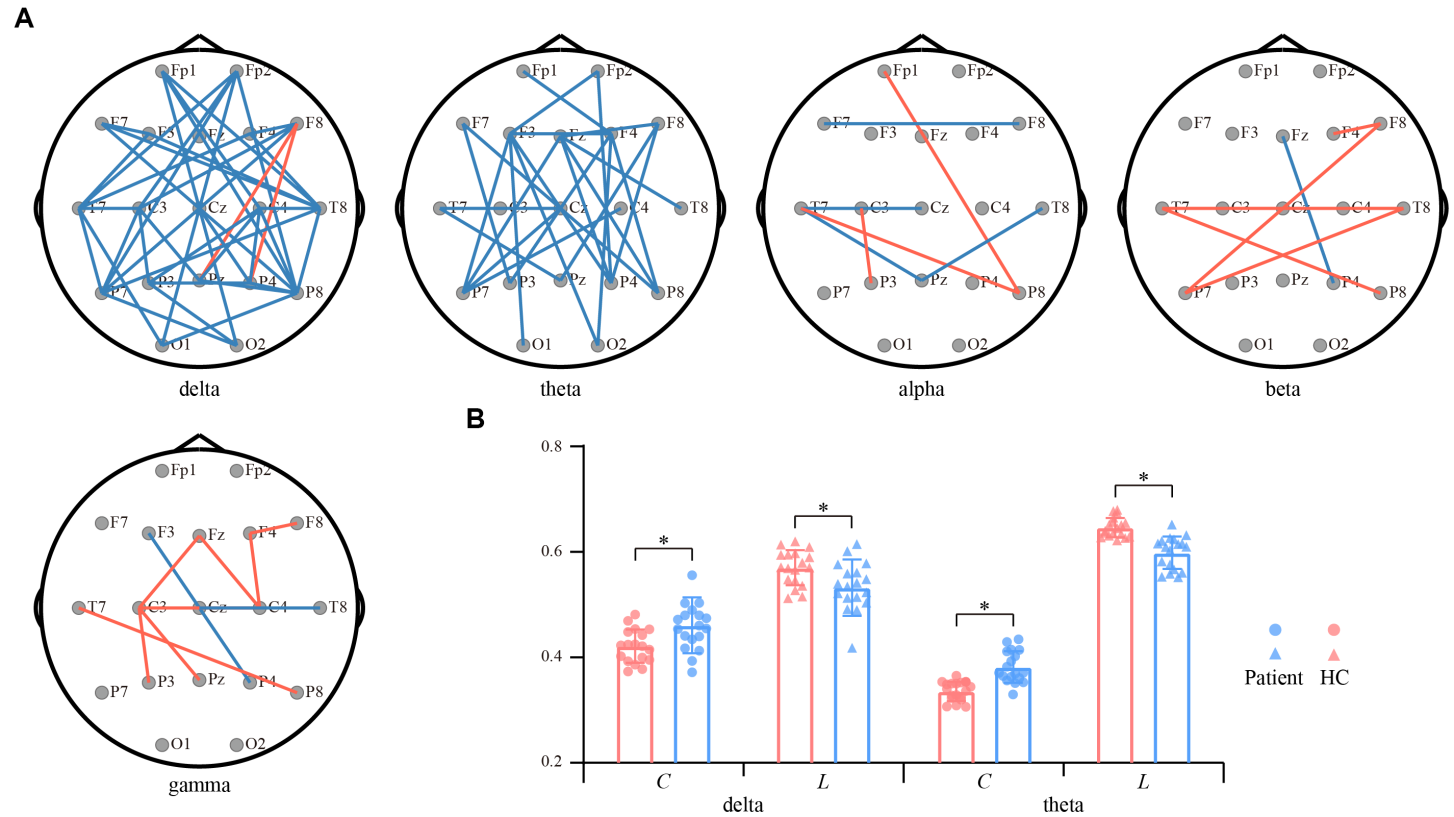
Differential resting-state networks between convalescent patients and HCs. **(A)** Differential network topologies within the five bands. Red and blue lines represent enhanced and suppressed connectivity of the patients compared to that of the HCs (*p* < 0.01, FDR corrected), respectively. **(B)** Differential network properties within the delta and theta bands. The red and blue bars represent patients and HCs, respectively. The symbol * represents *p* < 0.01.

In contrast to the *PSD*, functional network architectures for both interest bands (i.e., the delta and theta) show the opposite reorganized patterns (Figure 3A). Specifically, in the delta and theta bands, the PLV couplings spanning the frontal, temporal, and parietal lobes were attenuated for the convalescent anti-NMDARE patients in contrast to that of the HCs (*p* < 0.01, FDR corrected). Whereas, within the other bands, there only existed small portions of connections that exhibited significant differences. Figure 3B further shows the network properties of both bands, in which, the decreased *C* and longer *L* of the patients deviating from the HCs were found (*p* < 0.01), which coincided with the significantly reduced connections in Figure 3A.

### 3.3. Categorization into anti-NMDARE patients and HCs

**Figure 4:**
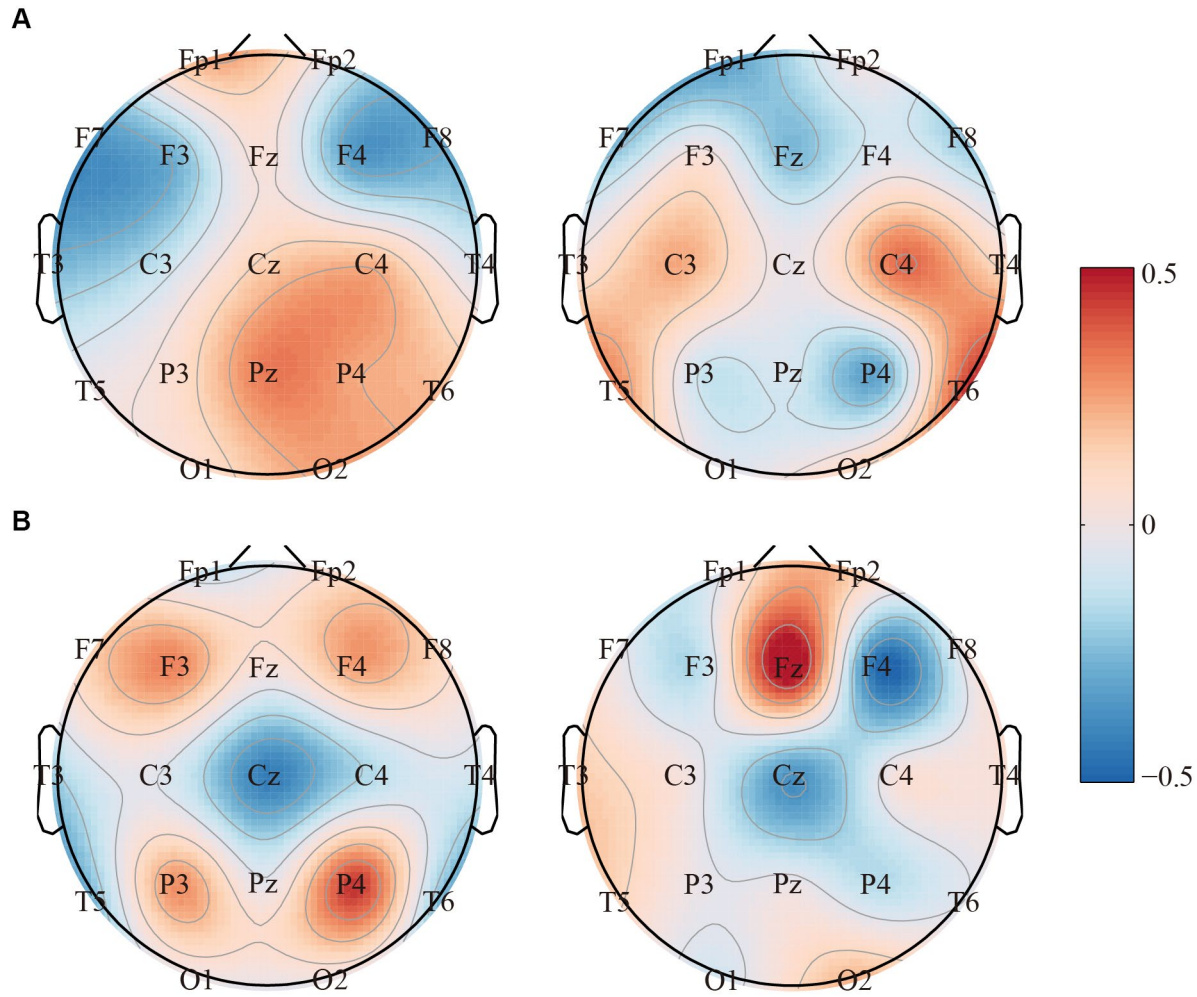
The topographies of the SPN filters for the **(A)** delta band and **(B)** theta band, respectively.

Given the significant differences identified above, we then investigated if these indexes could be regarded as biomarkers for anti-NMDARE identification. Thereinto, to capture the spatial network topologies, SPN was first applied to extract the inherent topological information. Notably, as exhibited in Figure 4, brain regions with significantly different network connections were highlighted with larger coefficients. Finally, by merging the *aPSD*, network metrics, and SPN features, we performed the classification of convalescent anti-NMDARE patients from HCs. Results show that an accuracy of 97.22%, specificity of 94.44%, and sensitivity of 100% were achieved by SVM. Namely, only one out of 36 subjects were falsely classified into the opposite groups, indicating a good classification performance.

## 4. Discussion

Up to now, the objective and quantified evaluation of brain deficits in convalescent anti-NMDARE patients has not been achieved accurately and noninvasively. Developing specific and objective biological markers is of great significance for the prognosis evaluation and discharge of this disease. Given the diagnostic value of EEG has received increasing attention in anti-NMDARE, herein, resting-state EEG of convalescent anti-NMDARE patients was recorded during the hospitalization and based on which, *SPT*s and network patterns were investigated to identify the reorganized and distorted brain rhythms, as well as develop potential biomarkers for distinguishing convalescent patients from HCs.

In this study, the rhythm-specific *SPT*s that reflect the regional distortion of EEG power in convalescent anti-NMDARE patients are given in Figure 2A. Concretely, within both delta and theta rhythms, the larger rhythmic power, as well as the increasing *aPSD*, were found in patients and centralized mainly in the middle and posterior brain regions. It appeared that patients with anti-NMDARE may experience disturbances in the brain rhythm even after undergoing treatment and recovering, especially for the delta and theta rhythms. Of note, the current results are globally in line with previous evidence showing that most patients have extensive EEG abnormalities characterized by generalized or focal slow delta-theta activity (32), which might be associated with a possible autoimmune etiology (33). As reported, delta oscillation is essential for the normal functioning of the human brain, and the increasing delta power has been widely documented in developmental disorders and pathological conditions, e.g., schizophrenia (34) and Alzheimer’s disease (35). Concerning the anti-NMDARE, the enhanced delta power in the NMDA-receptor antibodies positive model across a wide range of time-constant fluctuations has also been reported (36), while the weaker delta power thereby indicates a recovery in patients with encephalitis (37). Additionally, a higher delta peak is found to be associated with poorer clinical outcomes and thus indicates anti-NMDAR-mediated synaptic dysfunction (38). Considering theta activity is regarded as a credible EEG indicator of normal brain functions including attention and memory (39), and delta activity is involved in the integration of cerebral activity with homeostatic processes (40), the distortions of both rhythmic powers may thereby implicate the persistent and residual pathological states such as anhedonia or reward deficiency (40) in anti-NMDARE patients even after clinical recovery.

In contrast to the increasing EEG power, the attenuated network interactions that can quantify the dysfunctional resource allocations in these patients are depicted in Figure 3. In essence, previous studies have identified extensive impaired network connections in anti-NMDARE patients during the acute stage, including the visual, lateral-temporal, frontal–parietal, and sensorimotor networks (41, 42). Although these patients recruited in the current study had good therapeutical outcomes during convalescence, the inter-regional couplings within both delta and theta bands were still attenuated, in contrast to that of the HCs (Figure 3A). As proved in previous studies, the distorted frontal–parietal connections may relate to the psychotic symptoms (43), coinciding with the psychiatric model which relies on the frontal–parietal connectivity and NMDAR regulation (44). Additionally, abnormal frontal connectivity might also be associated with disrupted executive function in anti-NMDARE patients (45), while the attenuated occipital connectivity was assumed to be accompanied by reduced visual acuity that correlated with disease severity (46). Collectively, current observations may suggest the residual expression of NMDARs across the extensive brain regions and emphasize its persistent detrimental influence in convalescent patients.

Another possible interesting issue was about the opposite alterations between EEG power and functional networks. This is an expected result as the hyperactivated EEG power across extensive brain regions (Figure 2A) in turn obstructs the inter-regional information interaction, thereby resulting in the reduced functional connectivity in Figure 3A. Further, the disturbed network topologies were also quantitatively measured by the changing network properties of *C* and *L* (Figure 3B). As network properties directly measure the network efficiency (47), smaller *C* and longer *L* consistently illustrate the decreasing information processing capacity and efficiency of the brain, and might also index the impaired brain efficiency in convalescent anti-NMDARE patients. As such, the human brain works in a relatively balanced way to effectively process information, the rhythmic alterations in patients were governed by the need for information processing, which may reveal the underlying pathomechanism of the anti-NMDARE.

Thereafter, specifically investigating in Figure 4, we observed that the SPN filters can directly capture the topological differences between the two groups, as brain regions with differentiated interregional information interactions were marked with larger coefficients. In the meantime, given the significant rhythmic alterations in patients found above, features of EEG power and network patterns were further fused to distinguish convalescent anti-NMDARE patients from HCs. As illustrated, the performance further confirmed that these EEG features were feasible and might be reliable biomarkers to quantify the residual brain deficits in convalescent patients, as an accuracy of 97.22% (specificity of 94.44% and sensitivity of 100%) was achieved. These consistently reminded us that the resting-state EEG metrics might be the potential biomarker for future prognostic evaluation of convalescent anti-NMDARE patients, which could be the future direction for the following studies.

One possible limitation would be that the current analyses concentrated on the patients in the recovery phase, leaving the acute stage unmined. Therefore, in future works, patients in both the acute and recovery phases would be recruited, along with their EEG being collected. By performing related analyses, particularly the contrast between the two phases, the findings derived from our current study would be further validated.

## 5. Conclusion

This study investigated the rhythm-specific distortions of EEG power and network topologies in anti-NMDARE patients even after clinical recovery. We found oppositely configured patterns of EEG power and networks in the delta and theta bands, as the hyperactivated brain power obstructs the inter-regional information interactions in patients and further leads to attenuated frontoparietal and frontotemporal couplings. Importantly, merging EEG power and network features of delta and theta bands helped reliably differentiate the convalescent patients from HCs. Taken together, our observations provide new insights into the neural mechanism underlying the residual brain deficits in convalescent anti-NMDARE patients, and the distinguished resting-state EEG metrics are also expected to serve as the biomarkers for quantifying the therapy of anti-NMDARE, as well as providing potential clinical guidance for the future follow-up of these patients.

## Data availability statement

The raw data supporting the conclusions of this article will be made available by the authors, without undue reservation.

## Ethics statement

The studies involving human participants were reviewed and approved by the Ethics Committee of the First Affiliated Hospital, Zhejiang University School of Medicine. The patients/participants provided their written informed consent to participate in this study.

## Author contributions

DW and KW designed the study and wrote the protocol. LJ and BC managed the literature searches. LJ and RH performed the data analyses. LJ, BC, and RH undertook the statistical analysis. LJ and DW wrote the first draft of the manuscript, which was critically revised by FL, PX, KW, and DY. All authors contributed to and have approved the final manuscript.

## Funding

This work was supported by the National Natural Science Foundation of China (#U19A2082 and #62103085) and the Zhejiang Provincial Natural Science Foundation of China (#LY19H090020).

## Conflict of interest

The authors declare that the research was conducted in the absence of any commercial or financial relationships that could be construed as a potential conflict of interest.

## Publisher’s note

All claims expressed in this article are solely those of the authors and do not necessarily represent those of their affiliated organizations, or those of the publisher, the editors and the reviewers. Any product that may be evaluated in this article, or claim that may be made by its manufacturer, is not guaranteed or endorsed by the publisher.
